# Do the new ACG *Helicobacter pylori* treatment guidelines have implications for Canada?

**DOI:** 10.1093/jcag/gwaf008

**Published:** 2025-05-06

**Authors:** Sander Veldhuyzen van Zanten, Thomas Krahn

**Affiliations:** Division of Gastroenterology, Department of Medicine, University of Alberta, Edmonton, Alberta T6G 2X8, Canada; Division of Gastroenterology, Department of Medicine, University of Alberta, Edmonton, Alberta T6G 2X8, Canada

## Introduction

Recently the ACG published updated guidelines for the treatment of *Helicobacter pylori* infection.^1^ These new guidelines made significant changes in the choices for first line therapy. The question is whether these changes are relevant for Canada.

In 2016 and 2017, several updated treatment guidelines were published including the Canadian Association of Gastroenterology (CAG) Toronto consensus published in 2016, and American College of Gastroenterology (ACG) and the European Maastricht V guidelines.^2–4^ For the most part their recommendations were well aligned with each other. The main recommendations of the Toronto guidelines were:

1- that most treatments should have a duration of 14 days,
2- that a clarithromycin containing regimen should not be used, if a prior clarithromycin containing regimen had failed,
3- either concomitant (PPI–amoxicillin–metronidazole–clarithromycin; PAMC) therapy or bismuth-based quadruple therapy (BQT) were recommended first line therapies,
4- BQT or alternatively the combination of PPI, amoxicillin, and levofloxacin (PAL) were recommended as second and third line options,
5- the combination of PPI, amoxicillin, and rifabutin (PAR) was largely recommended as fourth line therapy.

## What the guidelines say

For treatment naïve patients the updated 2024 ACG guidelines recommend several first line options: bismuth-based quadruple therapy (BQT), rifabutin-based triple therapy (PAR), the potassium-competitive acid blocker (PCAB) Vonoprazan dual therapy with amoxicillin and Vonoprazan triple therapy with amoxicillin and clarithromycin, all for 14 days (Table 1). Importantly use of concomitant therapy (PAMC) was not recommended in treatment naïve patients with *H. pylori* infection.^1^

**Table 1.** Description of optimized treatment regimens used for *H. pylori* infection.

	Treatment	Medications	Dose used	Duration
Optimized regimens for H. pylori	Concomitant therapy (PAMC)	(1) Proton pump inhibitor (2) Amoxicillin (3) Clarithromycin (4) Metronidazole	1 tablet BID 1 g BID 500 mg BID 500 mg BID	14 days
	Bismuth-based Quadruple therapy (BQT)	(1) Proton Pump Inhibitor (2) Bismuth-subsalicylate (3) Metronidazole (4) Tetracycline	1 tablet BID 524 mg QID^a^ 500 mg QID 500mg QID	14 days
	PPI–amoxicillin–levofloxacin (PAL)	(1) Proton Pump Inhibitor (2) Amoxicillin (3) Levofloxacin	1 tablet BID 1 g BID 500mg BID^b^	14 days
	Rifabutin-based triple therapy (PAR)	(1) Proton Pump Inhibitor (2) Amoxicillin (3) Rifabutin	1 tablet BID 1 g BID 150 mg BID^c^	10 days
Commercially available combination pill preparations in the United States	Pylera	(1) Omeprazole (separate pill) (2) Bismuth subcitrate-K (3) Metronidazole (4) Tetracycline	20 mg PO BID 420 mg QID 375 mg QID 375 mg QID	10 days
	Talicia	(1) Omeprazole (2) Amoxicillin (3) Rifabutin	40 mg TID 1000 mg TID 50 mg TID	14 days
	Voquezna DualPaK	(1) Vonoprazan (2) Amoxicillin	20 mg BID 1 g TID	14 days
	Voquezna TriplePaK	(1) Vonoprazan (2) Amoxicillin (3) Clarithromycin	20 mg BID 1 g BID 500 mg BID	14 days

Please see notes below for doses that differed from the recommended doses by the Toronto consensus guidelines.

^a^Recommended dose of bismuth subsalicylate is 2 tabs of 262 mg PO QID.

^b^Recommended dose of levofloxacin is 250 mg PO BID.

^c^Recommended dose of rifabutin is 150 mg PO qd (or bid in Maastricht VI guidelines).

One of the main problems with treating *H. pylori* is the increasing rate of resistance, especially against clarithromycin and levofloxacin. The ACG 2024 guidelines cite rates of resistance of 22.2%-31.5% and 37.6% to clarithromycin and levofloxacin, respectively in *H. pylori* in the United States.^1^ The reasons for recommending bismuth quadruple therapy (BQT) as first line are the lack of evidence of superiority of concomitant therapy over BQT, the increasing rate of clarithromycin and metronidazole resistance in North America and globally, the lack of efficacy data from North America comparing different treatment options, and concerns about antibiotic stewardship.^1^

Importantly this is a commentary on the new ACG guidelines, not an evidence-based guideline. The ACG recommendations are not fully applicable outside the setting of the United States. In Canada, vonoprazan and combination tablet medications are not currently available (Table 1). Studies are needed comparing PAMC to BQT before concomitant therapy is discarded. A meta-analysis of 1810 treatment naïve patients showed no significant difference between PAMC (85.2%) and BQT (87.4%).^5^ Data on the efficacy of PAMC in Canada are scarce, but in our own study, the efficacy of PAMC in treatment naïve patients was 87%.^6^ The recently updated Maastricht VI guidelines also do list PAMC as a first line option.^7^

Bismuth quad therapy has stood the test of time, but its downsides are that the 4 times a day treatment is cumbersome, requires daily ingestion of many pills (*n* = 22) and has more side effects than PAMC. As well, tetracycline is not as commonly available, and has limited use in some populations such as women with the potential to become pregnant and patients with photosensitivity. With proper instruction most patients can complete the 14 days treatment. Hp-EuReG, a large consortium of European countries, documented in one of their studies that compliance with BQT is high, >97%, serious side-effects rare, .08%, and only 1.3% of patients could not complete the 14 days therapy.^8^ There is strong data that demonstrate that BQT is the best second line therapy as well.^2,7,9^

In some countries, including the United States and parts of Europe, a triple tablet is available for the combination of bismuth subcitrate, metronidazole, and tetracycline (Pylera) approved for a duration of 10 days. The efficacy of this medication was 90% as first line and as rescue therapy.^10–12^ The Maastricht VI guidelines favor a treatment duration of 14 days for the triple tablet combination although it acknowledges that it is uncertain whether there is a difference between 10 and 14 days.^7^ Importantly, there are no direct comparisons between standard BQT and 3-in-1 bismuth-containing single capsule regimens lasting 10-14 days. More studies are needed to better determine the optimum duration of BQT. Another point worth mentioning is that in Europe mainly bismuth subcitrate is used. This is also the bismuth salt in Pylera. In North America bismuth subsalicylate is mainly used. There are no head-to-head comparisons of bismuth subsalicylate and bismuth subcitrate, nor comparing the 3 in 1 tablet (Pylera) to bismuth subsalicylate based BQT.^8^

In the Toronto guidelines use of PPI–amoxicillin–rifabutin (PAR) was mainly reserved as a fourth line therapy, given for 10 days.^2^ Its success rate in Canada has been 62%.^13^ In our recent study when used as second-or higher order therapies the success rate of PAR given for 10 days was 50%-57% from 2007 to 2021 and better than PAL in those treated as second- or higher order therapies, using the high dose of rifabutin 150 mg twice a day.^6^

Since 2019 a combination tablet of omeprazole, amoxicillin, and rifabutin has become available in the United States, Talicia (Table 1). The success rate of this 14 days regimen given as first line in 2 RCTs used for registration in the United States was 84%.^14^ It was therefore listed by the ACG as an option for first line therapy. In the United States a course of Pylera costs $376 USD and Talicia $794 USD (data from Google).

Importantly in Canada to date rifabutin is a restricted drug and special authorization for its use is needed. The limited data on rifabutin resistance to date have shown very low levels of resistance.^15–17^ Rifabutin can cause myelotoxicity but when given for 10-14 days for *H. pylori* treatment this is rare.^1,2,16^ The new ACG guideline recommend that rifabutin triple therapy be given as a 14 day course,^1^ however studies are lacking whether therapy for 14 days is superior to 10 days.^14,16^

The ACG also listed the dual combination of vonoprazan 20 mg bid and amoxicillin 1 g t.i.d (Voquezna DualPak) or the triple combination (Voquezna TriplePak) of vonoprazan 20 mg, amoxicillin 1 g and clarithromycin 500 mg all given bid for 14 days as possible first line options. Vonoprazan is a potassium-competitive acid blocker (PCAB) that has more potent acid suppression than PPIs.^18^ In RCTs, mostly conducted outside North America and Europe, the *H. pylori* eradication rate was 84.7% for vonoprazan triple therapy and 78.5% for the dual therapy, compared to a success rate of 78.8% for lansoprazole together with clarithromycin and amoxicillin, a combination that is no longer recommended.^2,19^ Neither Vonoprazan nor the combination therapies are currently available in Canada and thus not further discussed.

Another combination that has been studied is high-dose dual therapy (double dose PPI, ≥3 g/day of Amoxicillin) combination. A meta-analysis of 4 RCTs evaluating high-dose dual therapy in patients with at least 1 failed treatment found an eradication rate of 81%.^20^ This is comparable to other recommended therapies.^20^ The PPI and amoxicillin dosing varied from 2 to 4 times a day. For this dual combination dosing frequency of amoxicillin 4 times daily improves the success rate: 87% for q.i.d versus 73% for b.i.d.^21^ There are no published data on this combination from Canada.

The levofloxacin combination of PAL was limited to second or third-line therapy in the Toronto guidelines because of high background resistance rates. In Edmonton, Alberta the success rate of PAL in patients who failed first line therapy was found to be low at 35%-38%.^6^ We therefore believe that PAR should be used as third line therapy. Updated guidelines also suggest that levofloxacin triple therapy should only be used if the patient is known to harbor a sensitive strain, citing lower success rates, increasing resistance and the FDA black box warning for tendinitis and tendon rupture.^1^

## Antibiotic resistance data and choice of PPI

Unfortunately, data on antibiotic resistance of *H. pylori* in Canada are scarce despite the fact that it is well established that resistance is an important predictor of treatment failure. To date resistance to amoxicillin (<1%), tetracycline (<1%-2%), and rifabutin (<1%) is low making these antibiotics good choices to use. For clarithromycin both primary (in treatment naïve patients) and secondary (in treatment experienced patients) resistance has increased over the years. The reported rates for primary Clarithromycin resistance during 1996-2006 (Halifax data, NWT data) were 17%-20%.^17^ Secondary clarithromycin resistance rates are much higher (33-80% 1996-2006 NS data, 83%-85% 2007-2021 Edmonton data). The same is true for levofloxacin. The primary resistance rate for levofloxacin was already 19% in the 1996-2006 period (NS data) and was 20% -69% (Edmonton data) during 2007-2021. It was higher for secondary levofloxacin resistance (Edmonton data reported an increase of 28% to61% from 2007-2015 to 2016-2021) (6). The dual resistance rate to both clarithromycin and metronidazole was 2/14 (14%) in newly diagnosed and 21/36 (58%) in previously treated patients in an Edmonton tertiary referral population from 2016-2021 (6) and found to be 9% in NWT.^17^ Presence of dual resistance is a strong predictor of failure of PAMC.^7^ Metronidazole resistance rates are high, 56%, but as mentioned this can be partially overcome with higher doses of metronidazole.^7^,^22–24^

It is important for clinicians to keep these resistance data in mind when choosing anti-*H. pylori* therapies. Ideally local data on resistance profiles of *H. pylori* are known but in reality this is seldom the case. *In the absence of having local resistance data, the best substitute is regular assessment of success rates of treated patients to document they conform to expected levels of success. It is strongly recommended that cure rates of prescribed anti-Helicobacter treatments are tracked.* In our Edmonton experience first line PAMC had a success rate of 87%, and the cumulative success rate of using PAMC, BQT (or vice versa first or second line), followed by PAL and PAR was 88%,^7^ but likely would be higher if PAR was used as third line therapy. This means that the large majority of patients will be cured of their Hp.^6^

The choice of which PPI to use is also important. Pantoprazole is the most commonly used PPI in Canada. Using a comparison to 20 mg omeprazole equivalents, pantoprazole 40 mg is one of the weaker PPIs.^25,26^ Esomeprazole 20 and 40 mg, Omeprazole 40 mg or rabeprazole 20mg are more potent.^24,25^ Hp-EuReG data show that by using more potent PPIs (such as omeprazole 40mg bid, esomeprazole 20 or 40mg bid or rabeprazole 20 mg bid) the cure rates are slightly higher: for BI quad standard or high dose PPI, not low dose, for PAMC higher success rates are only improved with high dose PPI.^7,12,26^ Rabeprazole or Esomeprazole at doses of 20-40 mg BID can provide high dose acid suppression and are less affected by CYP2C19 metabolism than other PPIs.^27^

## What is the bottom line?

H. *pylori* treatment is complex and treatments must remain practical, accessible, and effective even in the absence of reliable resistance testing or available combination tablets. In Canada, both BQT and PAMC can be used as first line therapy. PAMC is easier for patients to take but BQT over the years has been a robust therapy. PAMC should not be used in patients who have failed a clarithromycin therapy or are known to harbor a clarithromycin resistant strain. If PAMC is used first and the patient is not cured BQT is the best second line therapy. If BQT was used as first line is PAMC a good second choice? Yes, unless the patient is known to have a clarithromycin resistant strain or has been exposed to clarithromycin. We no longer believe PAL should be third line therapy, unless one knows that the strain is levofloxacin sensitive. Instead, PAR should be used as third line. Using high dose PPI slightly improves the success rate of existing therapies. More studies are needed in North America before high-dose dual therapy of PPI and amoxicillin can be considered a routine treatment.

It is important to keep in mind that with each failed treatment the subsequent success rate will be lower than when used in a higher order treatment, and routine tracking of success rates of treatment can be helpful at minimizing antibiotic overuse even in the absence of resistance testing. Patients benefit from receiving a detailed handout explaining the importance of *H. pylori* infection, and providing instructions on when the different treatments should be taken. *Single pill formulations may ultimately simplify H. pylori treatment but are not currently available in many parts of the world, including Canada.* It is our practice to write all our prescriptions as blister packs to facilitate adherence. *We believe more data including studies in community-based settings are needed including comparisons to BQT and PAMC before PAR is listed as first line therapy.*

## Supplementary Material

gwaf008_suppl_Supplementary_ICMJE_Forms

## Data Availability

There are no data associated with this manuscript.
